# Do Gravity-Related Sensory Information Enable the Enhancement of Cortical Proprioceptive Inputs When Planning a Step in Microgravity?

**DOI:** 10.1371/journal.pone.0108636

**Published:** 2014-09-26

**Authors:** Anahid H. Saradjian, Dany Paleressompoulle, Didier Louber, Thelma Coyle, Jean Blouin, Laurence Mouchnino

**Affiliations:** 1 Aix-Marseille Université, CNRS, Laboratoire Neurosciences Cognitives UMR 7291, Marseille, France; 2 Fédération de Recherche 3C Comportement-Cerveau-Cognition, CNRS -Aix-Marseille University, Marseille, France; 3 Aix-Marseille Université, CNRS, Institut des Sciences du Mouvement, UMR 7287, Marseille, France; University of Bologna, Italy

## Abstract

We recently found that the cortical response to proprioceptive stimulation was greater when participants were planning a step than when they stood still, and that this sensory facilitation was suppressed in microgravity. The aim of the present study was to test whether the absence of gravity-related sensory afferents during movement planning in microgravity prevented the proprioceptive cortical processing to be enhanced. We reestablished a reference frame in microgravity by providing and translating a horizontal support on which the participants were standing and verified whether this procedure restored the proprioceptive facilitation. The slight translation of the base of support (lateral direction), which occurred prior to step initiation, stimulated at least cutaneous and vestibular receptors. The sensitivity to proprioceptive stimulation was assessed by measuring the amplitude of the cortical somatosensory-evoked potential (SEP, over the Cz electrode) following the vibration of the leg muscle. The vibration lasted 1 s and the participants were asked to either initiate a step at the vibration offset or to remain still. We found that the early SEP (90–160 ms) was smaller when the platform was translated than when it remained stationary, revealing the existence of an interference phenomenon (i.e., when proprioceptive stimulation is preceded by the stimulation of different sensory modalities evoked by the platform translation). By contrast, the late SEP (550 ms post proprioceptive stimulation onset) was greater when the translation preceded the vibration compared to a condition without pre-stimulation (i.e., no translation). This suggests that restoring a body reference system which is impaired in microgravity allowed a greater proprioceptive cortical processing. Importantly, however, the late SEP was similarly increased when participants either produced a step or remained still. We propose that the absence of step-induced facilitation of proprioceptive cortical processing results from a decreased weight of proprioception in the absence of balance constraints in microgravity.

## Introduction

Movement, in particular those whose control relies on sensory feedback can improve the transmission of sensory inputs that are known to be gated prior to and during a voluntary movement [1]–[7]. Indeed, cortical responsiveness to sensory stimuli can be increased during the execution of voluntary movements by alleviating the gating of sensory inputs to suit task-specific demands [8]–[13]. In addition, the amount of signal transmitted to the cerebral cortex is not uniform over the execution of a voluntary movement and can be dynamically modulated while the movement is being performed. For instance, following lower limb nerve stimulation during the different phases of locomotion, Altenmüller et al. [14] and Duysens et al. [15] showed that the attenuation of cutaneous afferents was less pronounced in anticipation of the foot contact as compared to the early swing phase. Interestingly, Duysens et al. [15] have shown that this increased sensory transmission was associated with an increased perception of tactile stimuli applied to the lower limb, presumably as a means to prevent loss of equilibrium at heel strike.

More recently, enhanced cortical activity was observed during the performance of lower limb movements requiring high level of accuracy (i.e., increasing task demands) [16],[17],[18]. For instance, analysing the cortical response evoked by the stimulation of proprioceptive inputs from the lower limb, we observed greater cortical response (i.e. sensory facilitation) when the stimulation occurred during the planning of gait initiation than when participants stood still without producing a step [18]. This facilitation can refer either to an enhancement of signal transmission or to an enhanced cortical processing. Nevertheless, in our previous study [18], the earlier component of the somatosensory-evoked potential (SEP P1-N1) could reffer at least partly, to signal transmission whereas the late task-specific facilitation (∼200 ms post-stimulation) could refer to a cortical processing [8]–[10] and not be related to the mere incoming sensory inputs. These findings extend to the planning phase of a voluntary movement the modulation of the cortical transmission reported during movement execution by Altenmüller et al. [14] and Duysens et al. [15]) and support the idea that the importance of the proprioceptive inputs varies online over the planning of complex motor behaviours such as gait initiation. One explanation for the sensory facilitation during the planning phase of a step execution may be related to the proprioceptive-based online control of equilibrium during the planning phase of a step execution. Indeed, this late step-related proprioceptive facilitation was not observed in microgravity environment in which equilibrium constraints are absent [18].

However, in microgravity one cannot disentangle whether the absence of facilitation mechanisms is due to the fact that proprioceptive inputs are irrelevant or simply not functional preventing the facilitation to be evoked. Indeed, in the former (i.e., irrelevant) facilitation mechanisms can be of no use because of the fact that they are linked to the planning of anticipatory postural adjustments (i.e., forces exerted onto the ground to shift the body weight prior to step movement), which are not observed in microgravity [19]. Alternatively, in the latter, such facilitation mechanisms could also be non-functional because of the decreased sensitivity to proprioceptive inputs. This hypothesis is supported by the large depression of the cortical response to the vibration in weightlessness compared to normogravity [18] and also by the findings that vibration-induced postural responses and kinaesthetic illusions are considerably reduced in weightlessness [20].

The origin of this proprioceptive impairment is still a matter of debate. Studies have suggested that it could result from the absence of gravity-based sensory inputs [21]–[24]. For instance, Clément and Lestienne [25] observed in astronauts, a large body tilt and the impossibility of maintaining a vertical posture. In addition, Parker et al. [26] observed again on astronauts, modifications of perceived self-motion during sinusoidal roll, which had been correctly interpreted as a pure roll in a pre-flight condition, but as a translation motion immediately post-flight (i.e., otolith tilt-translation reinterpretation hypothesis). The non-functionality of proprioceptive inputs could also be responsible for the larger errors in goal-directed movements observed in weightlessness [21],[27],[28].

In this framework, multisensory signals are processed to be transformed into common, whole-body centered, reference frames (for instance by the posterior parietal cortex, see for reviews Stein [29] and Pfeiffer et al. [30]). For example, Hlavačka et al. [31] showed an amplification of the lateral lean when combining lower leg muscle vibration and galvanic vestibular stimulation highlighting the role of vestibular inputs in establishing a reference system for the body proprioceptive inputs. Additionnaly, Karnath [32] found in neglect patients that an interactive effect of both vibratory proprioceptive and vestibular caloric stimulations contributes to the participants' mental representation of egocentric references. Using positron emission tomography, Bottini et al. [33] found evidence of neural correlates for this egocentric representation in the secondary somatosensory cortex, the temporo parietal junction and the perisylvian cortex.

However, such remapping of multisensory body-related signals could still provide ambiguous information on the body position relative to the external world (i.e., vertical posture and/or gravity). Horizontal or vertical linear acceleration relative to a support surface registered by either feet mechano- or vestibular receptors, could help resolving the ambiguity of proprioceptive information to estimate body position in space. Indeed there is substential evidence to support the hypothesis that exteroceptive cutaneous and/or vestibulo-based information relative to the horizontal and/or vertical orientation in space, together with proprioceptive inputs generate sensory percepts of the body position in space [34]–[36].

Here, we tested whether re-establishing a body-in-space reference frame in microgravity can enable the proprioceptive facilitation during the planning phase of a step movement as is seen in normogravity. To this end in microgravity, we stimulated the participants' cutaneous and vestibular receptors by translating the platform on which they were standing before they produced a step. The existence of a proprioceptive facilitation was assessed by analysing and comparing the cortical responses evoked by the vibration of the leg muscles (known to activate the muscle spindles [37]) with those recorded when no step had to be produced.

## Materials and Methods

### Ethics statement

This experiment was approved by the flight testing center. All participants gave their written informed consent to take part in this study, which conforms with the standards set in the Declaration of Helsinki. The local Ethics Committee (Sud Méditerranée 1, ID RCB: 2010-A00074-35) specifically approved the study.

### Experimental procedures

Six participants without any known neurological and motor disorders participated in the experiment (mean age 36±12 years). The experiment was conducted in the A-300 ZEROg aircraft chartered by the French Centre National d'Etudes Spatiales (CNES) for parabolic flight studies. Six flights over two parabolic flight campaigns (#VP95 and #VP98) were necessary to complete the experiment. During the flight, the aircraft alternated rises (acceleration) and descents (deceleration) to carry out parabolic profiles, which were interspersed with flat trajectories (constant velocity). Each parabolic maneuver was composed of three distinct phases: 20 s of hypergravity (1.8 g, pull-up phase) followed by 22 s of microgravity (0 g or µG), before a final 20 s period of hypergravity (1.8 g, pull-out phase). The aircraft performed a sequence of 30 parabolas per flight separated by 2- to 8-min periods of level flight. The microgravity phase provided enough time to perform 3 experimental trials per parabola and thus giving a total of 90 trials for each subject. To prevent free floating in the experimental bay, the participants wore shoes with adapted metal soles and stood on a platform comprising of four electromagnets (i.e., two under each foot). Each set of electromagnets could be de-activated independently allowing the release of the right foot 200 ms before the imperative signal for movement execution. At the start of the trials, the participants received the instruction of either to make a step forward with the right leg (i.e., Stepping condition) at an imperative tone signal or to stand still (i.e., Standing condition). The participants were asked to close their eyes upon receiving the instructions and keep them closed until the end of recording trial (i.e. 3 seconds).

### Stimulation procedures

**Figure 1:**
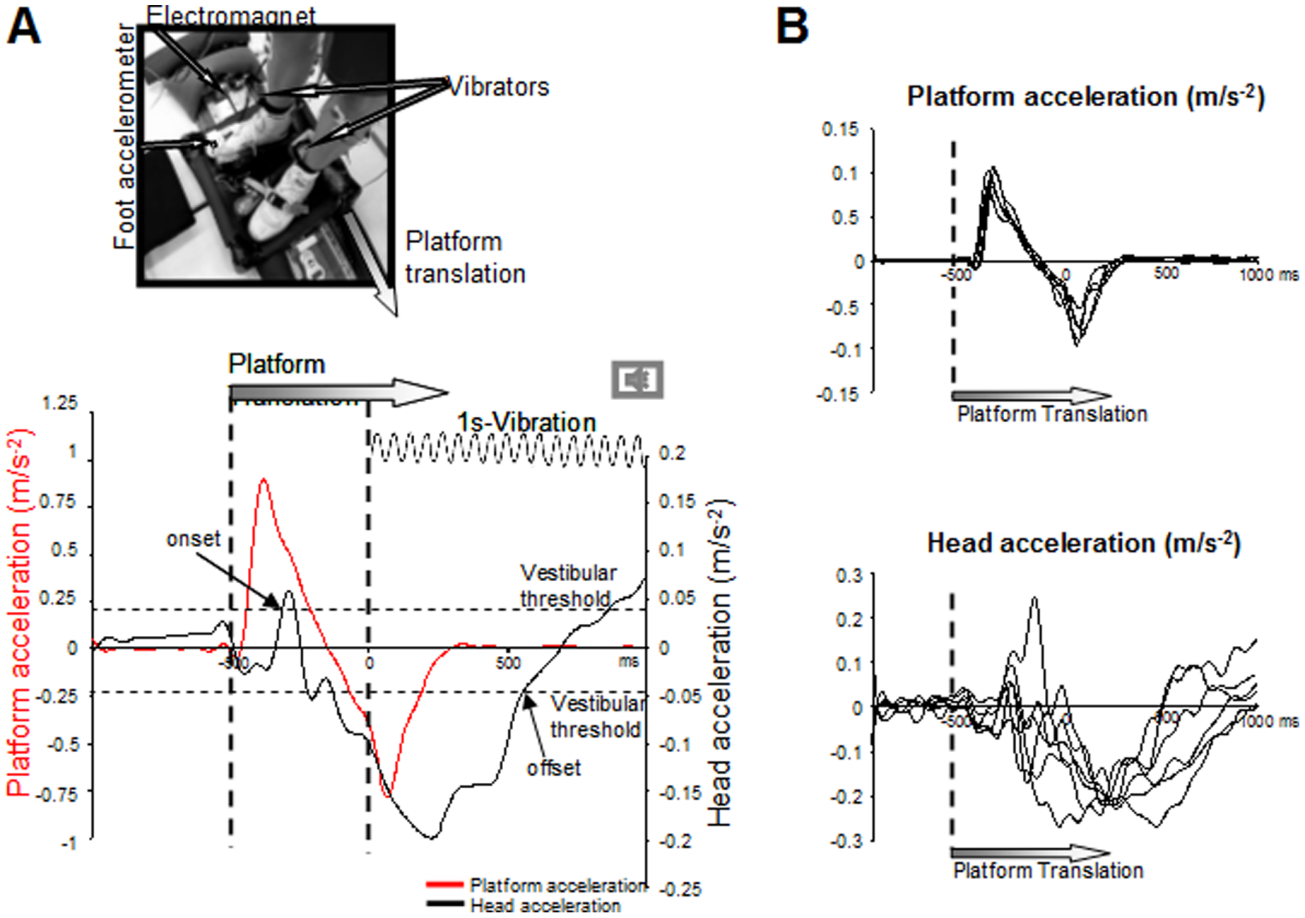
Experimental set-up and mean lateral platform acceleration (left scale and red curve) and head acceleration (right scale and black curve) during Translation standing condition for the 6 participants. The onset and offset of head acceleration and deceleration, respectively, were indicated by the arrows according to the vestibular threshold (horizontal dotted lines). **B:** Mean platform and head accelerations for each of the 6 participants.

In all the trials performed by the participants a 1-second vibration was applied on the ankle muscles. The vibration started ∼1 sec after the verbal cue to either stand still or execute a step. The vibration onset constituted a pre-cueing signal for the step initiation and the imperative signal/tone for step execution was synchronized with the end of the vibration. Specifically, tendons of the peroneus longus (stepping leg) and tibialis posterior (supporting leg) muscles (Fig. 1A) were vibrated simultaneously. These muscles are responsible for moving the ankle joints laterally and primarily sense leftward lateral body tilts. The vibratory stimulus consisted of small-amplitude vibrations (1.2 mm) of high frequencies (80 Hz), which are known to produce micro-stretches of muscle spindles and are subsequently interpreted by the central nervous system as resulting from muscular stretching [37]–[39].

In order to induce body-in-space related afferent signals, we translated the platform on which the participants were standing (Fig. 1). These translations stimulated somatosensory (e.g., cutaneous afferents, induced by the force applied to the skin of the feet by the moving platform with at least some contribution of somatosensory afferents from the feet and the ankle joint) and vestibular receptors. This platform was attached to a cable on the left side and to an electromagnet on the right side maintaining it stationary. The cable was run laterally through a spring system such that when the electromagnet was switched off, the platform moved of 10 cm slightly to the left reaching a mean peak acceleration of 0.78 m.s^2^±0.21 (Fig. 1A, red curve). Importantly, the onset of the head acceleration (above the vestibular threshold: reported as “onset”) occurred later (Fig. 1A, black curve).

The ankle muscles were vibrated in all three experimental conditions. In the Stationary standing condition, the platform was maintained stationary during the whole trial duration. In both the Translation standing and Translation stepping conditions, the platform was displaced laterally 500 ms before the vibration. The platform translation lasted on average ∼800 ms and it was therefore stationary when the participants had to produce a step at the imperative tone signal (i.e., Translation stepping). Each participant performed 30 trials per condition (total of 90 trials) and the conditions were presented in random order.

### Behavioural recordings and analyses

The kinematics of the stepping movement was recorded using a triaxial accelerometer (Analog device) placed on the top of the right foot (Fig. 1A). Vertical acceleration of the foot was analyzed for the sole condition where a step was required (i.e., Translation stepping), to determine the onset of the step. This was found to occur 321 ms±14 after the tone signal and reached a peak acceleration of 1.76±0.65 m.s^2^.

Head acceleration was measured by using a triaxial accelerometer (Model 4630: Measurement Specialties, USA) placed on the chin. The head acceleration and its latency with respect to the platform translation onset were analyzed in the lateral direction (i.e, direction of the platform displacement, Fig. 1B). We determined the head acceleration onset as the time when the acceleration reached the vestibular threshold (set at 0.048 m.s^2^ by Gianna et al. [40]) and the offset as the time when the head deceleration returned to sub- vestibular threshold in a monotonic way. Maximal head acceleration was also analysed.

Bipolar surface electromyography (EMG, Bortec AMT- 8 system: Bortec Bomedical, Canada) was used to record the activity of the tibialis anterior (TA) and of the gastrocnemius medialis (GM) muscles of both legs. Activations of the TA and GM muscles of the stepping leg are responsible for the shift the body weight prior to step movement [19],[41],[42]. EMG signals were pre-amplified at the skin site (×1000), sampled at 1000 Hz, band-pass filtered 20 to 250 Hz and rectified. In order to quantify the muscle activity, we computed the integral of the EMG activity (iEMG) for each muscle during two time-windows identified from electroencephalographic measures, as described below.

### Electroencephalographic recordings and analyses

Electroencephalographic (EEG) activity was recorded continuously from 64 Ag/AgCl surface electrodes embedded on an elastic cap (BioSemi ActiveTwo system: BioSemi, Netherlands). Specific to the BioSemi system, “ground” electrodes were replaced by Common Mode Sense active and Driven Right Leg passive electrodes. The signals were pre-amplified at the electrode sites and post-amplified with DC amplifiers, filtered on-line with a 0.16 Hz high pass filter (Actiview acquisition program) and digitized at a sampling rate of 1024 Hz. Signals from each channel were referenced using the average of the 64 scalp electrodes. They were further filtered off-line with 50 Hz notch filters (digital filters, 24 dB/octave), 48 Hz (high cut-off) and 0.1 Hz (low cut-off) filters (digital filters, 48 dB/octave; BrainVision Analyzer 2, Brain Products, Germany). Vertical electro-oculograms were recorded bipolarly with electrodes placed above and below the left eye; horizontal electro-oculograms were recorded bipolarly with electrodes positioned near the outer canthus of each eye. EEG signals were corrected for eye blinks according to the statistical method of Gratton et al. [43], as implemented in the BrainVision Analyzer 2 software.

Somatosensory evoked potentials (SEPs) were obtained by averaging, for each subject and each condition, all synchronized epochs relative to the stimulus onset (either vibration or platform translation onsets). The average amplitude of the 200-ms pre-stimulus epoch served as baseline. For the two Translation conditions (standing and stepping), the baseline was set before the translation onset). After visual inspection of the EEG traces, 5% of trials had to be rejected due to artefacts.

**Figure 2:**
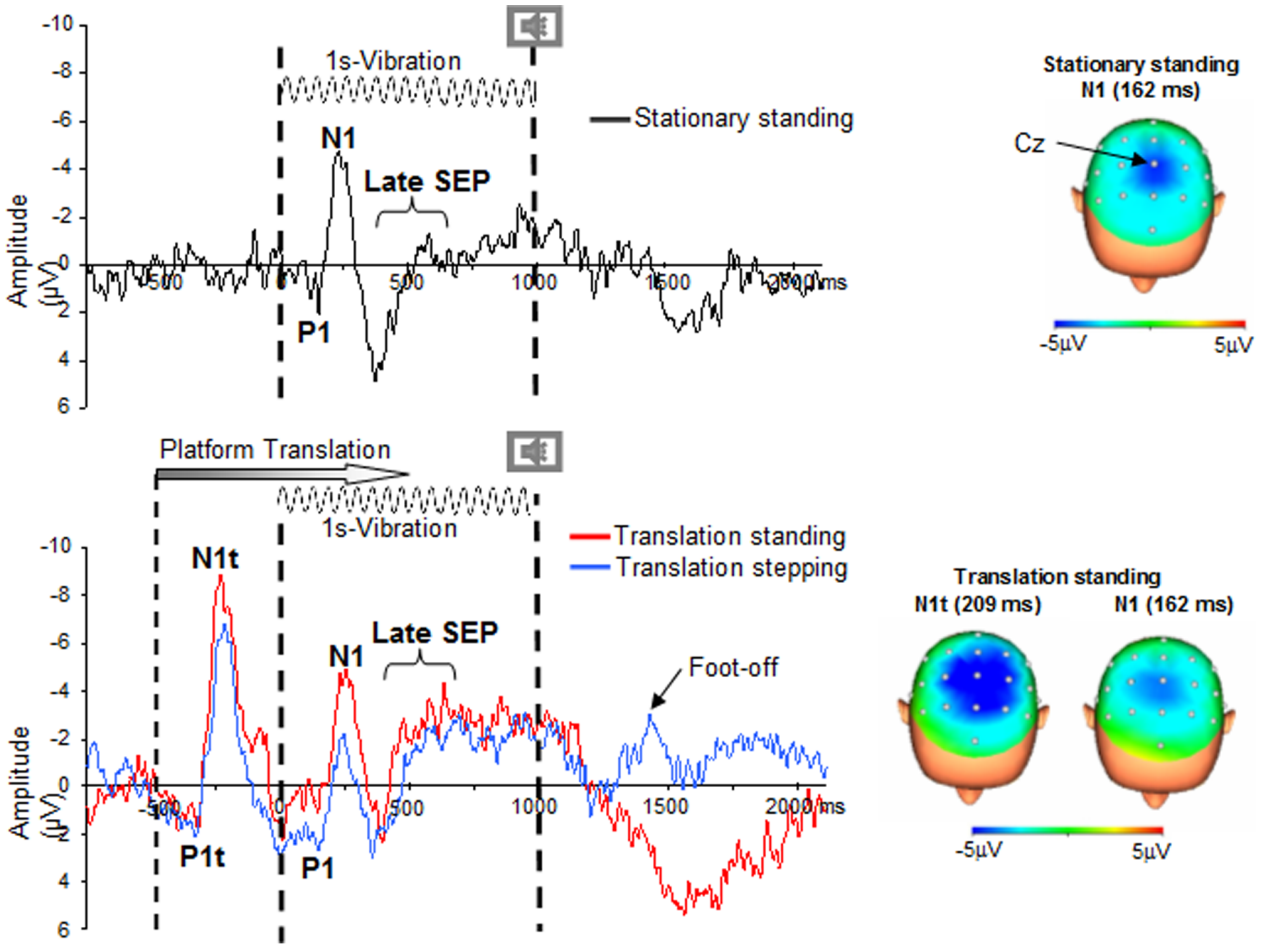
SEPs recordings. Grand-Average for 6 participants recorded at electrode Cz for the Stationnary standing condition (Top panel), Translation standing and Translation stepping conditions (Bottom panel). The vertical dotted lines indicate the vibration onset and offset, the second vertical dotted line also indicates imperative tone stimulus for step execution. For both Translation conditions, the vertical dash-dotted line indicates the translation onset (occurring 500 ms before vibration). The “foot-off” indicates the onset of the stepping movement computed on the foot vertical velocity. The scalp topography was shown at the peak negativity for the participants average in the Sationary and Translation conditions.

The SEPs were found to be maximal at the Cz electrode in all conditions (Fig. 2). EEG analyses were conducted on the activity recorded at this electrode which overlays the somatosensory cortices. We primarily focused on the P1-N1 complex following the sensory stimulation evoked by the vibration or the P1t-N1t complex evoked by the translation (t) when present (Fig. 2). Its amplitude was measured peak-to-peak and the latency of its components was measured as the time of the first positive (P1 or P1t) or negative (N1 or N1t) peaks relative to stimulation onset.

Following the P1-N1 complex, a clear negative wave rose over the somatosensory cortices (referred to as the late SEP) We quantified this late SEP activity by computing the integral of the EEG activity (iEEG) over the interval elapsed between the first opposite deflection after N1 and 600 ms after stimulation onset. Behavioural iEMG analyses were performed during the time-window defined from P1 onset to opposite deflection after N1 and during the late SEP.

### Statistical analyses

All dependent variables showed normal distributions (i.e. p>0.05, Shapiro-Wilk tests). The EEG data were submitted to one-way repeated measures analysis of variances (ANOVAs) having 3 levels (Stationary standing, Translation standing, Translation stepping). Behavioural data relating to muscular activity were submitted to repeated ANOVAs combining 3 conditions (Stationary standing, Translation standing, Translation stepping) with 2 Sides (Left, Right). All significant main and interaction effects are reported for all analyses. Significant effects were further analysed using Newman-Keuls post-hoc tests. Behavioutal data related to head accelerations were submitted to paired t-tests comparing the 2 Translation conditions (Standing and Stepping). The level of significance was set at 5% for all analyses.

## Results

### Early SEP evoked by the platform translation (P1t-N1t)

The earliest peak discernible in the EEG traces after the platform translation was a positivity (i.e., P1t) which was followed by a large negativity (N1t) (Fig. 2 bottom panel). The t-tests did not show significant difference between the Standing and Stepping conditions neither for P1t (average = 142±23 ms) nor for N1t latencies (average = 209±20 ms) (t_5_ = 1.16; p = 0.29 and t_5_ = 0.10; p = 0.92, for P1t and N1t, respectively). As for latencies, the P1t-N1t SEP amplitude was not significantly different between Standing and Stepping conditions (overall mean 10.22 µV±3.65, t_5_ = −2.42; p = 0.06).

To determine the timing between the somatosensory and the vestibular stimulations induced by the platform translations, we computed the time elapsed before the acceleration of the head reached the vestibular threshold (set at 0.048 m.s^2^ by Gianna et al. [40]) after translation onset (considered as the onset of the somatosensory stimulation). On average, head acceleration reached the vestibular threshold (Fig. 1A) 329±150 ms after the translation onset (no significant difference between Translation standing and Translation stepping conditions, t_5_ = −0.19; p = 0.856).

The large lag between the onsets of the platform translation which did not show variability in the timing across participants (see Fig. 2B), and head-in-space motion could be explained by the considerable whole body inertia. Indeed the inertia of the body could have played a damping role, thus delaying and limiting the head response to the platform acceleration. As it occurred before acceleration of the head reached the vestibular threshold, the P1t-N1t complex was most likely evoked by somatosensory afferent inflow of the feet during platform translation rather than by vestibular inputs. Before reaching the vestibular threshold, the early head response to platform translation was rather consistent across participants (see Fig. 2B); afterwards as the body stiffness could be quite different for each participant, the maximal head acceleration was also quite different.

Afterwards the head reached a peak acceleration 712±166 ms long after the platform translation (Fig. 1A) which did not significantly differ between both translation condition and was on average 0.097±0.036 m.s^2^ (t_5_ = −0.18; p = 0.85).

We computed the duration of head acceleration and deceleration to assess if vestibular afferents were present during the late somatosensory process. The head acceleration-deceleration lasted on average 861±116 ms (t_5_ = 1.05; p = 0.34) indicating that the offset of head deceleration occured ∼300 ms before the imperatif tone signal for movement execution (i.e., offset of vibration). This suggests that vestibular afferents together with somatosensory afferents were present during the late SEP.

### Early SEP evoked by vibration

**Figure 3:**
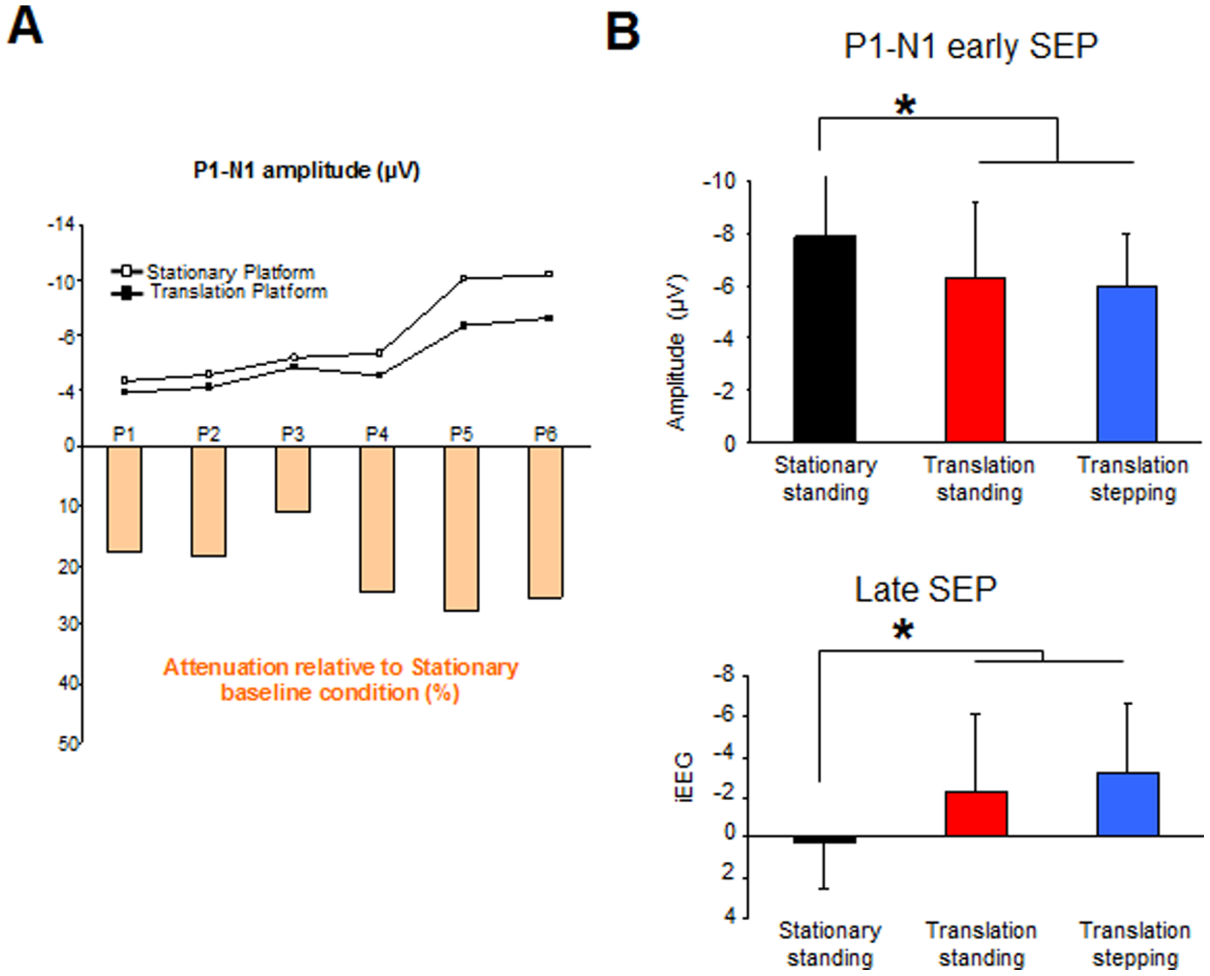
Mean P1-N1 SEP amplitude of each participant (P1–P6) for the Stationary and Translation platform conditions (upper panel). Normalized attenuation for the translation condition relative to the Stationary condition (bottom panel). The mean attenuation for the 6 participants was of 21% (±6). **B:** Mean amplitudes for 6 participants of the P1-N1 early SEP and mean integral of EEG activity (iEEG, late SEP) computed in a time window comprising between early SEP ending until 600 ms (*: p<0.05).

After vibration onset, the earliest discernible peak was a positivity (i.e., P1) which had an average latency of 94±19 ms and which was followed by a large negativity (i.e., N1) whose peak occurred on average 162±16 ms after vibration onset (Fig. 3). The ANOVA did not show a condition effect either for P1 or for N1 latencies (F_2, 10_ = 1; p = 0.39 and F_2, 10_ = 3.39; p = 0.07, for P1 and N1, respectively). While these latencies were not affected by the prior platform translation (i.e., somatosensory stimulation), the amplitude of the P1-N1 complex was significantly depressed by the translation (Fig. 3, F_2,10_ = 4.50; p = 0.04). The amplitude was larger in the Stationary standing condition (7.91±3.43 µV) than in both translation conditions (6.26±2.93 µV and −6.005±1.97 µV in Translation standing and in Translation stepping, respectively, Fig. 3A) whose P1-N1 amplitudes were not significantly different (p = 0.71).

The muscle activity (i.e., iEMG) computed during the P1-N1 SEP did not show any significant effect for both condition (F_2,10_ = 1.77; p = 0.21) and side (left and right, F_1,5_ = 0.76; p = 0.42).

### Late and sustained negative waveform (i.e late SEP)

**Figure 4:**
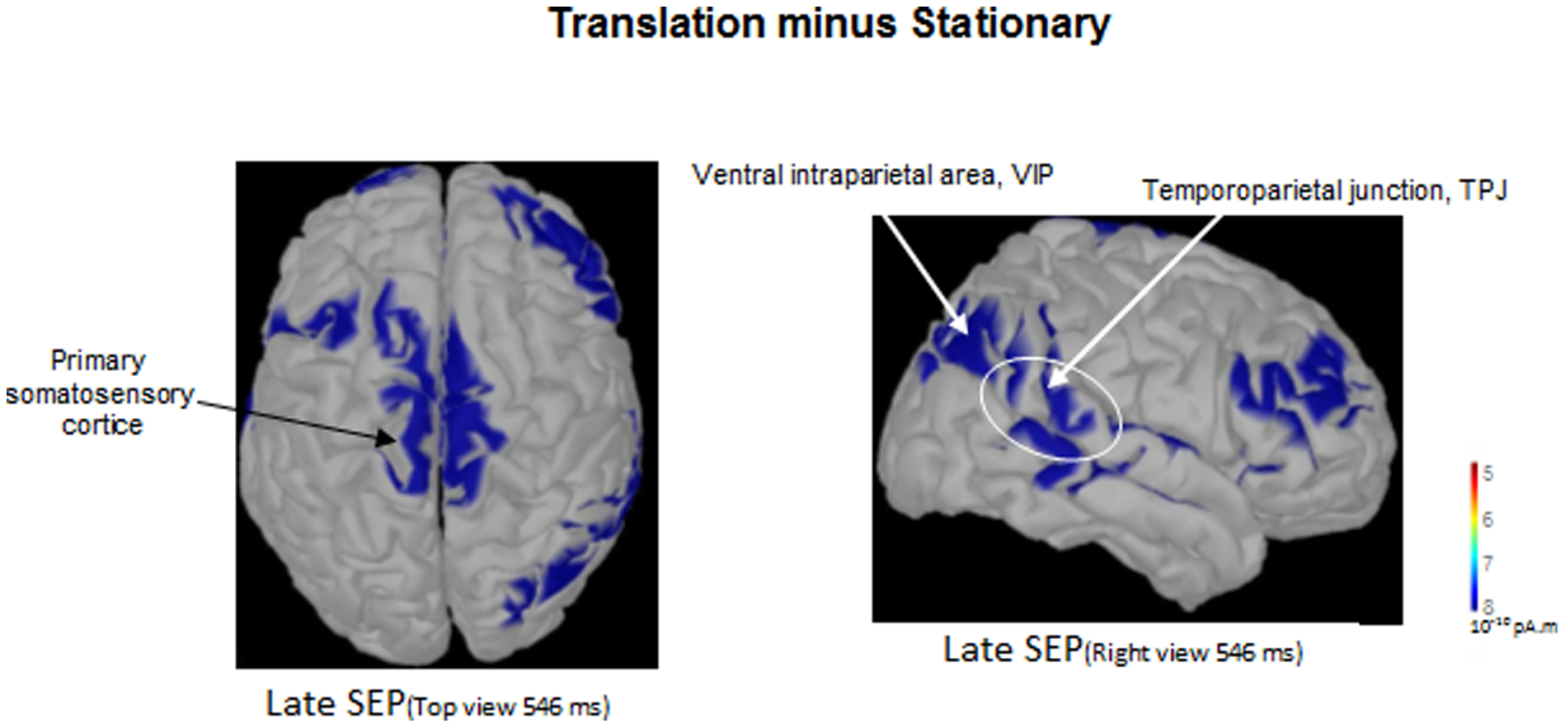
Results of source reconstruction from the grand average EEG data of the 6 participants (Low Resolution Electromagnetic Tomography, sLORETA) displayed on the used source space (Montreal warp brain aligned to the co-ordinate system of Talairach and Tournaux). sLORETA images depicted the estimated current density strengh corresponding to the effect of translation relative to baseline (Translation minus Stationary) for the Late SEP. The scale of the maps was chosen to maximize identification of the sources and were given a marked threshold to only show source activity that was 18% upper of minimal activation. Note the clearly distinguishable activation above the primary somatosensory cortice (top view) and the right posterior parietal cortex (e.g., VIP) and the Temporoparietal region.

A late and sustained negative waveform developed over the somatosensory cortices after the P1-N1 component (Fig. 2). This wave started to develop 283±32 ms relative to the vibration onset and its latency did not differ between condition (F_2,10_ = 0.015; p = 0.98). The ANOVA showed a main condition effect on the iEEG (F_2,10_ = 6.87; p = 0.01); the iEEG during late SEP was smaller in the Stationary standing condition (0.97±2.70 µV) than in both Translation standing (p = 0.039) and Translation stepping (p = 0.011) conditions (Fig. 3B). Interestingly, in both Translation conditions, post hoc analyses did not show differences between Standing and Stepping (P = 0.22) (1.46±4.69 µV and −2.79±4.24 µV for the Translation standing and Translation stepping conditions, respectively). As shown by the source reconstruction (Low Resolution Brain Electromagnetic Tomography, sLORETA) from the grand average EEG data (Fig. 4), brain activity computed from the Translation minus Stationary conditions differed during the late component. Importantly, the effect of translation relative to baseline (i.e., Translation minus Stationary, Fig. 4), showed electrical sources above the primary somatosensory cortices together with right hemisphere sources (not observed in the left hemisphere). As the right posterior cortical regions (e.g., parieto-insular vestibular cortex, PIVC and ventral intraparietal area, VIP) are involved in the processing of vestibular signals (evoked by head acceleration), it is not surprising to find theses sources (Fig. 4, Right view) together with the somatosensory source (Fig. 4, top view). This lateralized activation observed over the right hemisphere might be due to lateralized stimulation (i.e., left translation of the platform).

The muscle activity during the late SEP was not significantly different between conditions (F_2,10_ = 1.74; p = 0.22) and side (F_1,5_ = 4.53; p = 0.086).

## Discussion

Stimulation of cutaneous and vestibular receptors is considered as a mean to enhance reference systems which are used in sensorimotor processes, particularly in the absence of gravity-based sensory inputs as in weightlessness [21]–[24]. Here, we tested whether establishing a reference system with similar sensory stimulation in microgravity would give rise to a facilitation of the proprioceptive input during the step planning similar to that identified in normogravity but not seen in the absence of gravity [18]. In the current sudy we found that cutaneous and vestibular stimulations increased the late cortical response evoked by the subsequent proprioceptive stimulation (induced by vibration of leg muscles). However, this sensory facilitation was observed irrespectively of whether or not participants were planning a stepping movement during the proprioceptive stimulation. These results argue for a lack of task-specific demands for processing proprioceptive input when planning a step in microgravity.

### Sensory interference phenomenon

The first notable result of the present experiment was the depression of the vibration-related early SEP following the platform translation. This depression may correspond to the so-called “sensory interference phenomenon” that was reported by Burke et al. [44]–[46]. A sensory interference is known to be induced when the stimulation of a given sensory modality (here, Ia afferents) result in a smaller evoked potential when it is preceded by the stimulation of a different sensory modality (here, feet cutaneous and vestibular afferents due to the platform translation together with some contribution of proprioceptive signals in the ankles, hips and upper part of the body). Therefore, the increased afferent activity from somatosensory (i.e., mainly cutaneous and proprioceptive receptors) and vestibular receptors would have attenuated the early cortical activity evoked by the vibration (busy line). This busy-line phenomenon, which has been reported frequently in studies of the somatosensory system, may be explained by refractoriness in the peripheral nerves themselves (Morita et al. [6]), by depression of synaptic transmission from the primary afferents (Hultborn et al. [47]), or by interference anywhere along the ascending sensory pathway and in the cortex itself. While the previous studies have revealed the existence of such interference for inter-stimulus intervals of 300 ms [6], the present findings show that the busy-line phenomenon can still be observed for inter-stimulus as long as 500 ms. Because the amplitude of the vibration-induced P1-N1 complex was attenuated by the preceding platform translation irrespective of whether participants had to plan a stepping movement or not (i.e., Translation stepping and Translation standing conditions) precluded the possibility that the sensory attenuation resulted from cortical processes related to movement planning.

### Potentiation phenomenon of combined stimulations independent of task requirement

A key finding of the present study is that the late SEP was significantly greater when the platform on which the participants were standing moved slightly before vibration of the leg muscles compared to when the platform remained stationary. One may argue that the late component corresponded to a return to baseline. However, based on our previous paper [18] that showed that the late component is modulated depending on the task to perform (i.e., Standing or Planning a step) and on the present data (i.e., difference between Stationary standing condition and both Translation conditions), the late component increase was likely task- or context-dependent. In addition, because the late cortical activation was observed above the primary somatosensory cortices and also in the right posterior cortical regions (e.g., PIVC and VIP regions) which process vestibular signals and spatial aspect of bodily self-consciousness ([30] for review), it is unlikely that the late component resulted from the mere return to baseline in both Translation conditions.

This late facilitation may also stem from cognitive processes such as those linked to the fear of falling. Indeed, it is known that cognitive and attentional functions can influence balance and postural control [48] and that the activity of the somatosensory cortex increases when expecting postural perturbations [49]–[52]. Because cognitive factors are known to influence the late SEP [53],[54], covert/overt attention may have contributed to the late negative wave increase observed here.

It is worth noting that the late activity increased in both conditions with platform translations despite the depression of the early P1-N1 component observed under these same conditions. This suggests that the sensory interference phenomenon affected only the initial afferent volley to the cortex and not the later SEP component which is thought to reflect higher order, integrative processing stage of somatosensory input [9],[10],[54]. The combined cutaneous and vestibular stimulation may have provided a reference system which enhanced the processing of the subsequent proprioceptive inputs. Such a hypothesis was proposed by Carriot et al.'s [28] after observing that feet cutaneous stimulation in microgravity improved the subject's perception of body orientation. Kinematic analyses of the platform-induced movement of the head showed that head acceleration was above vestibular threshold during the late SEP. The facilitation of the proprioceptive integrative process observed with platform translations is therefore in line with the recent finding by Ferrè et al. [55],[56] showing facilitation of somatosensory input by vestibular (caloric) stimulation. Cells from widely distributed brain regions have been found to integrate vestibular and somatosensory inputs and could have contributed to the increased SEP recorded here. Among these cells are those of the parieto-insular vestibular cortex which is considered an important node for multisensory integration [57]–[60]. This area is known to be involved in the whole body experience built up from multisensory integration [61] and has dense connections with the somatosensory cortex [62]. Behavioural studies have also highlighted such potentiation processes when combining lower leg muscle vibration and galvanic vestibular stimulation in postural control [63],[64] and during locomotion [65]. A representative example of somatosensory and vestibular interaction was also highlighted by Horak and Hlavačka [66] who showed on patients with somatosensory loss caused by peripheral neuropathy, an increased vestibulospinal sensitivity.

More importantly, however, despite the greater integration of proprioceptive inputs allowed by the cutaneous and vestibular stimulations, the planning of stepping movements did not lead to greater sensory facilitation compared to the condition where participants remained still after the platform translation. This is in large contrast with the increased late SEP found in normogravity when preparing similar stepping movements [18]. Therefore, the enhancement of the late SEP observed in both translation conditions (Standing and Stepping) most likely corresponded to a non-specific multisensory integration process that had little or no contribution to the planning of stepping movements. This is in line with Kennedy and Inglis [67], whose study shows that the potentiation phenomenon does not appear specific to balance task requirements (e.g. step initiation) as it was also observed in human participants adopting a prone posture when combining galvanic vestibular stimulation (GVS) and tibial nerve stimulation.

In this light, the absence of late facilitation of proprioceptive input when participants planned stepping movements could be explained by the absence of equilibrium constraints in microgravity rather than by a reduction of proprioceptive input in weightlessness. We hypothesized that facilitation mechanisms were of no use because of the fact that they are linked to the planning of anticipatory postural adjustments (progressively assembled and stored well before (i.e., 1.5 s) being triggered, [68]) which are not observed in microgravity [19]. Overall, the present study, in line with others [27],[69],[70] strongly suggests that gravitational influences are taken into account for limb (arm or leg) movement organization and execution in a predictive manner. For instance, it was shown that removing gravity affects slow movements (more feedback-driven) more than fast ones (more dependent on centrally generated activity, [27],[69]). It has been further hypothesized that gravity is encoded in the central nervous system and that the cerebellum may contain an internal representation of gravitational torques used for sensorimotor predictions [71].
